# The complete mitochondrial genome of *Orancistrocerusaterrimusaterrimus* and comparative analysis in the family Vespidae (Hymenoptera, Vespidae, Eumeninae)

**DOI:** 10.3897/zookeys.790.25356

**Published:** 2018-10-15

**Authors:** Qiao-Hua Zhang, Pan Huang, Bin Chen, Ting-Jing Li

**Affiliations:** 1 Institute of Entomology & Molecular Biology, College of Life Sciences, Chongqing Normal University, Chongqing 401331, China Chongqing Normal University Chongqing China

**Keywords:** Eumeninae, mitochondrial genomes, *
Orancistrocerus aterrimus aterrimus
*, phylogenetic analysis, Vespidae

## Abstract

To date, only one mitochondrial genome (mitogenome) in the Eumeninae has been reported in the world and this is the first report in China. The mitogenome of *O.a.aterrimus* is 17 972 bp long, and contains 38 genes, including 13 protein coding genes (PCGs), 23 tRNA genes, two rRNA genes, a long non-coding region (NCR), and a control region (CR). The mitogenome has 79.43% A + T content, its 13 PCGs use ATN as the initiation codon except for *cox1* using TTG, and nine genes used complete translation termination TAA and four genes have incomplete stop codon T (*cox2*, *cox3*, *nad4*, and *cytb*). Twenty-two of 23 tRNAs can form the typical cloverleaf secondary structure except for *trnS1*. The CR is 1 078 bp long with 84.69% A+T content, comprising 28 bp tandem repeat sequences and 13 bp T-strech. There are two gene rearrangements which are an extra *trnM2* located between *trnQ* and *nad2* and the *trnL2* in the upstream of *nad1*. Within all rearrangements of these mitogenomes reported in the family Vespidae, the translocation between *trnS1* and *trnE* genes only appears in Vespinae, and the translocation of *trnY* in Polistinae and Vespinae. The absent codons of 13 PCGs in Polistinae are more than those both in Vespinae and Eumeninae in the family Vespidae. The study reports the complete mitogenome of *O.a.aterrimus*, compares the characteristics and construct phylogenetic relationships of the mitogenomes in the family Vespidae.

## Introduction

Animal mitochondrial genomes (mitogenomes) have been widely used in studies of molecular evolution, population genetic structure, and phylogeny because of their stable gene content, rapid evolutionary rate, relatively conserved gene arrangement, maternal inheritance, and infrequent recombination (Wolstenholme 1992; Saccone et al. 1999; Oliveira et al. 2008; Li et al. 2017). The family Vespidae has more than 5000 known species worldwide, which are divided into six subfamilies, Euparagiinae, Masarinae, Eumeninae, Stenogastrinae, Polistinae, and Vespinae (Carpenter 1993), but their phylogenetics have not been settled. There have been ten mitogenomes sequences reported in the Vespidae (seven in the subfamily Vespinae, three in Polistinae, and one in Eumeninae) (Table 1). Among these six subfamilies, there are more than 3600 species in the subfamily Eumeninae worldwide, more than half of the known species of Vespidae. The species in Eumeninae, also known as potter wasps, are solitary, and mostly catch caterpillars as food for their next generation in the environment of farmlands, forests, and orchards, which can directly control caterpillar pests. To date, there is only one species (*Abispaephippium*) with its mitogenome published (Cameron et al. 2008). *Orancistrocerusaterrimusaterrimus*, the species under study in this work, belongs to the Eumeninae, and is widely distributed in China (Jiangsu, Anhui, Fujian, Jiangxi, Hunan, Guangxi, Chongqing, Sichuan, Yunnan provinces), and Laos, Vietnam (Li 1985; Selis 2018).

In the present study, the complete mitogenome of *O.a.aterrimus* was sequenced using Illumina sequencing technique, and its characteristics analyzed, including gene rearrangements, nucleotide composition, codon usage, etc. More importantly, the phylogenetic relationships of 12 species of mitogenomes in Vespidae are constructed and discussed based on nucleotide sequences of 13 PCGs using both Maximum Likelihood (ML) and Bayesian Inference (BI) methods. The study updates phylogenetic research based on the mitogenomes, and provides basic information framework of mitogenomes in Vespidae for further research on the phylogenetic relationships of both genera and subfamilies in this family.

**Table 1.** The information of Vespidae mitogenomes used in the phylogenetic analysis in the present study.

**Subfamily**	**Species**	**Migenome size (bp)**	**Gene number**	**GenBank Accession**	**Reference**
**Ingroup (Vespidae)**					
Eumeninae	* Orancistrocerus aterrimus aterrimus *	17972	38	KY941926	This study
Eumeninae	* Abispa ephippium *	16953	41	EU302588	Cameron et al. (2008)
Polistinae	* Polistes jokahamae *	16616	34	KR052468	Song et al. (2016)
Polistinae	* Polistes humilis synoecus *	14741	34	EU024653	Cameron et al. (2008)
Polistinae	* Parapolybia crocea *	16619	37	KY679828	Peng et al. (2017)
Vespinae	* Vespula germanica *	16342	33	KR703583	Zhou et al. (2016)
Vespinae	* Vespa ducalis *	15779	37	KX950825	Kim et al. (2017a)
Vespinae	* Vespa mandarinia *	15902	37	KR059904	Chen et al. (2015)
Vespinae	* Vespa bicolor *	16937	35	KJ735511	Wei et al. (2014)
Vespinae	* Vespa velutina nigrithorax *	16475	37	KY091645	Kim et al. (2017b)
Vespinae	* Vespa orientalis *	16101	37	KY563657	Nizar et al (2017)
Vespinae	* Dolichovespula panda *	17137	37	KY293679	Fan et al (2017)
**Outgroup (Formicidae)**					
Formicinae	* Formica selysi *	16752	37	KP670862	Yang et al. (2015)

## Materials and methods

### Sample collection and DNA preparation

The specimens of *O.a.aterrimus* were collected from Yangshuo county of Guangxi province, preserved in the 100% ethanol, and stored at -20 °C. Total DNA of a single adult specimen was extracted from the muscle tissues using the DNeasy DNA Extraction Kit (QIAGEN) in accordance with the manufacturer’s instructions. The concentration of genomic DNA in extraction product was assayed on a Qubit fluorometer using a dsDNA High-sensitivity Kit (Invitrogen).

### Mitogenomes sequencing and assembling

The Illumina TruSeq library was constructed from the gDNA with the average length of the inserted fragment of 480 bp. The library was sequenced on a full run of Illumina Hiseq 2500 with 500 cycles and paired-end sequencing (250 bp reads). High-quality reads were used in *de novo* assembly with IDBA-UD after removing adapters, unpaired, short and low quality reads (Peng et al. 2012). With IDBA-UD, these parameters have a similarity threshold of 98% and minimum and maximum k values of 80 and 240 bp, respectively. To identify the mitogenome assemblies from the pooled sequencing files, two different fragments of mtDNA (*cox1* and *rrnS*) were amplified as bait sequences by standard PCR reactions using primers designed with reference of Simon et al. (2006). Using BLASTN search against the reference of bait sequences, matching rate of 100% was confirmed as the mitogenome of *O.a.aterrimus*. The identical or near-identical overlapping terminal regions of mitogenome sequences were examined and circularized by Geneious (http://www.geneious.com/).

### Sequence annotations and analysis

PCGs and rRNA genes were aligned with other published Vespidae insect mitogenomes by Clustal X (Thompson et al. 1997). The majority of the tRNA gene locations and secondary structures were identified by tRNAscan-SE Search Server v.1.21 (Lowe and Eddy 1997), and the remaining tRNA were identified in comparison with other known species of tRNAs in Vespidae (Cameron et al. 2008; Song et al. 2016). The CRand the tandem repeat sequence were analyzed with Tandem Repeats Finder (http://tandem.bu.edu/trf/trf.html) (Benson 1999). Base composition and codon usage in all 12 mitogenomes of Vespidae were calculated by MEGA v 6. 0 (Tamura et al. 2013). In addition, the AT skew = [A - T] / [A + T] and GC skew = [G - C] / [G + C] were computed (Perna and Kocher 1995).

### Phylogenetic analysis

Eleven known mitogenome sequences in the family Vespidae and the mitogenome sequence of *Formicaselysi* (KP670862) in the family Formicidae were downloaded from GeneBank, and that of *O.a.aterrimus* was produced in the present study (Table 1). The phylogenetic tree of 12 mitogenomes sequences in the family Vespidae was constructed using ML and BI methods with MEGA 6.0 (Tamura et al. 2013) and MrBayes 3.1.1 (Huelsenbeck and Ronquist 2001), and the *Formicaselysi* (KP670862) was used as outgroup. The nucleotide sequences of 13 PCGs were applied in the phylogenetic inference, and the best fitting substitution model was detected using Mrmodeltest 2.3 (Nylander 2004). The bootstrap values were calculated based on 1000 replications, and the confidence values of the topology is high.

## Results and discussion

### Genomic organization

**Figure 1.**
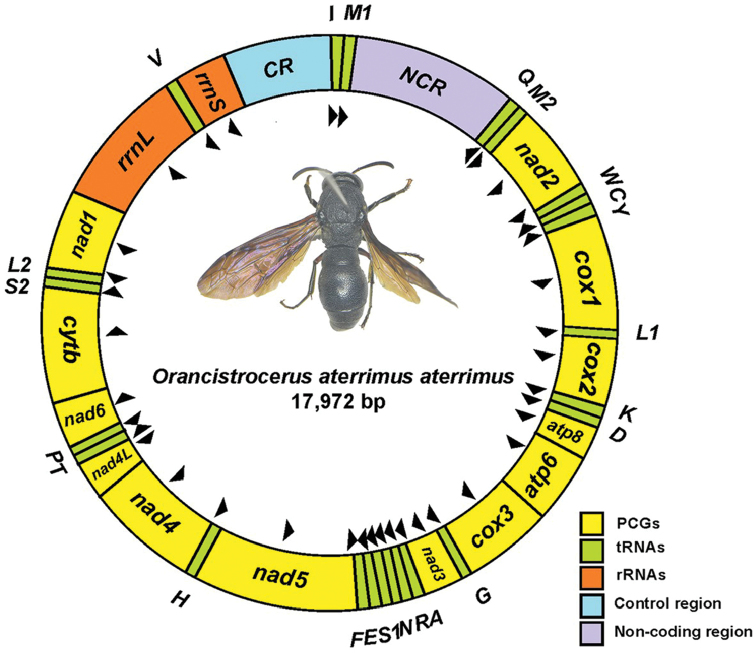
The mitochondrial genome of *O.a.aterrimus*. Arrows indicate the direction of genes. Abbreviations of the gene name are as follows: *nad1-4* and *nad4L* act as nicotinamide adenine dinucleotide hydrogen dehydrogenase subunits 1-6 and 4L; *cox1*, *cox2*, and *cox3* act as the cytochrome C oxidase subunits; *cytb* act as cytochrome b; *atp8* and *atp6* act as adenosine triphosphate synthase subunits 6 and 8; *rrnL* and *rrnS* act as large and small rRNA subunits; In addition, *CR* indicates control region and *NCR* indicates non-coding region.

The complete mitogenome of *O.a.aterrimus* is a double-strand of circular molecular DNA and 17,972 bp. It contains 38 genes: 13 PCGs, 23 tRNAs, two rRNAs, a control region (CR), and a long non-coding region (NCR) (Figure 1), of which 24 genes are situated in the majority strand (J-strand) and the other 14 genes are located in the minority strand (N-strand) (Table 2). An extra *trnM2* and a long NCR were found in the mitogenome. The gene *trnM2* is 67 bp and located in 2 142-2 208 between *trnQ* and *nad2*. The NCR is 1 946 bp long, located in 128-2 073 between *trnM1* and *trnQ*. With the exception of the NCR (1 946 bp), 14 intergenic spacers exist and sum to 174 bp, of which the longest spacer is 48 bp long, located between *nad4l* and *trnT*. In addition, a total of 24 bp overlaps was identified in 12 genes, with the overlap length of each gene ranging from 1 to 8 bp.

**Table 2.** Mitochondrial genome annotation of *O.a.aterrimus*.

**Gene**	**Direction**	**Location**	**Size (bp)**	**Anticodon**	**Codon**		**Intergenic nucleotides**
					**Start**	**Stop**	
*trnI*	F	1–63	63	30–32 GAT			
*trnM1*	F	63–127	65	93–95 CAT			-1
non-coding region							1946
*trnQ*	R	2074–2138	65	2108–2110 TTG			0
*trnM2*	F	2142–2208	67	2173–2175 CAT			3
*nad2*	F	2209–3234	1026		ATC	TAA	0
*trnW*	F	3249–3315	67	3280–3282 TCA			14
*trnC*	R	3308–3374	67	3342–3344 GCA			-8
*trnY*	R	3383–3447	65	3416–3418 GTA			8
*cox1*	F	3446–4981	1536		TTG	TAA	-2
*trn L1*	F	5006–5073	68	5035–5037 TAA			24
*cox2*	F	5074–5752	679		ATC	T-	0
*trnK*	F	5753–5824	72	5785–5787 CTT			0
*trnD*	F	5824–5893	70	5858–5860 GTC			-1
*atp8*	F	5894–6049	156		ATC	TAA	0
*atp6*	F	6049–6720	672		ATG	TAA	-1
*cox3*	F	6742–7525	784		ATG	T-	21
*trnG*	F	7526–7593	68	7556–7558 TCC			0
*nad3*	F	7594–7947	354		ATT	TAA	0
*trnA*	F	7947–8011	65	7977–7979 TGC			-1
*trnR*	F	8011–8074	64	8038–8040 TCG			-1
*trnN*	F	8078–8147	70	8108–8110 GTT			3
*trn S1*	F	8147–8206	60	8168–8170TCT			-1
*trnE*	F	8214–8277	64	8244–8246 TTC			7
*trnF*	R	8277–8342	66	8307–8309 GAA			-1
*nad5*	R	8344–10032	1689		ATT	TAA	1
*trnH*	R	10033–10096	64	10065–10067 GTG			0
*nad4*	R	10097–11402	1306		ATA	T-	0
*nad4l*	R	11399–11677	279		ATT	TAA	-4
*trnT*	F	11726–11789	64	11756–11758 TGT			48
*trnP*	R	11789–11858	70	11823–11825 TGG			-1
*nad6*	F	11860–12399	540		ATG	TAA	1
*cytb*	F	12403–13534	1132		ATG	T-	3
*trnS2*	F	13544–13612	69	13572–13574 TGA			9
*trnL2*	R	13640–13707	68	13676–13678 TAG			27
*nad1*	R	13708–14676	969		ATA	TAA	0
*rrnL*	R	14682–16044	1363				5
*trnV*	R	16043–16106	64	16074–16076 TAC			-2
*rrnS*	R	16107–16894	788				0
Control region		16895–17972	1078				0

### Gene rearrangements

**Figure 2.**
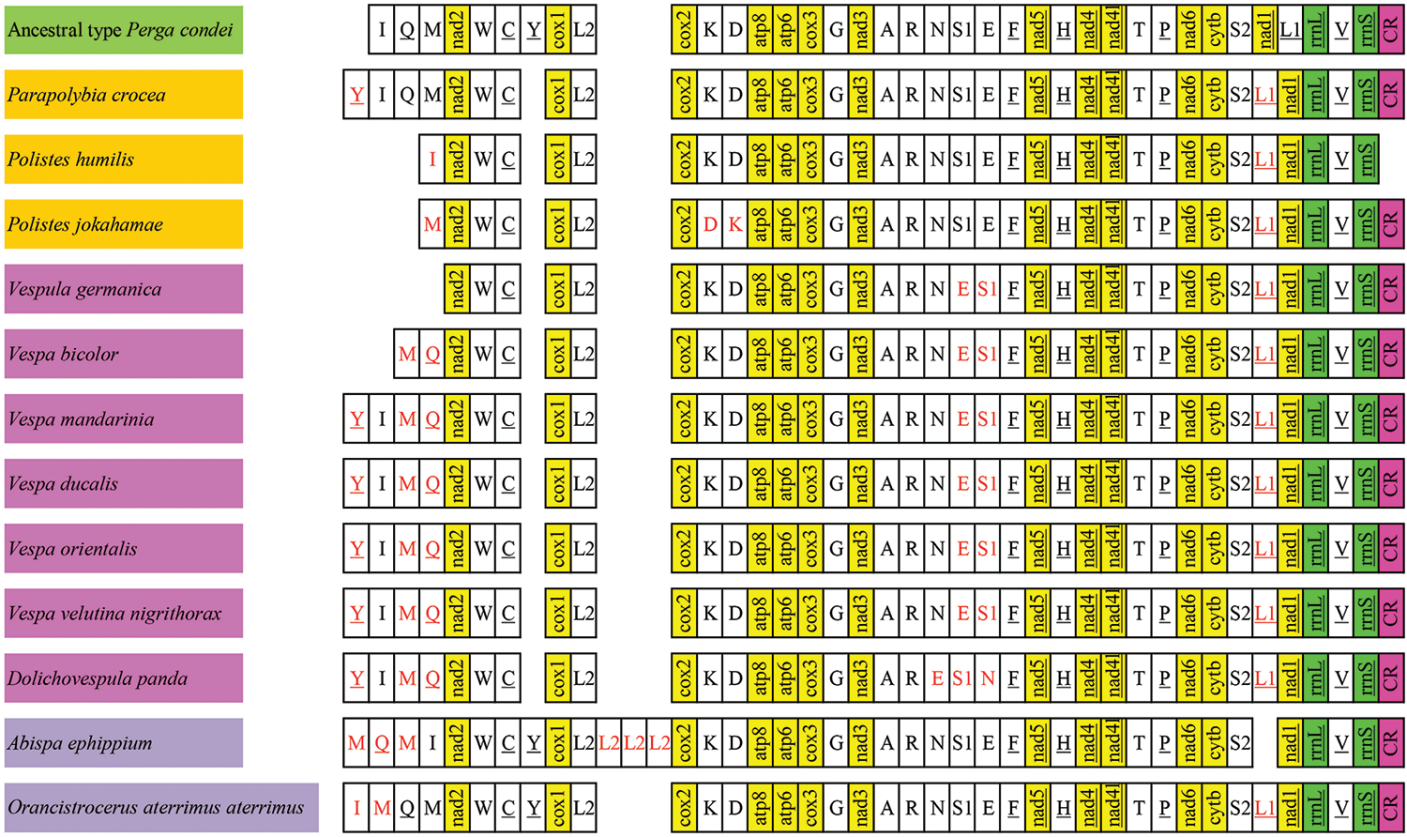
Mitochondrial gene arrangement of 12 species of Vespidae. The red fonts indicate the rearrangement of the genes.

The gene order of 13 PCGs and two rRNAs in *O.a.aterrimus* mitogenome is consistent with the putative hymenopteran ancestor: the sawfly *Pergacondei* (Hymenoptera: Symphyta: Pergidae:) (Castro and Dowton 2005). However, there are two rearrangements of tRNAs in the mitogenome (Figure 2), namely, an extra *trnM2* and *trnL2* in the upstream of *nad1*, contributing to the novel gene order: *trnL2 - nad1 - rrnL - trnV - rrnS - CR - trnI - trnM1 - trnQ - trnM2 - nad2* (Figure 2). In the mitogenome of *Abispaephippium*, another species in the subfamily Eumeninae, the gene order of rearrangements is *trnL2 - trnM1 - trnQ - trnM2 - trnI, trnL1 - trnL1 - trnL1 - trnL1* and *trnS2 - nad1* (Figure 2) (Cameron et al. 2008). In the subfamily Polistinae, the translocation between *nad1* and *trnL1* is present in three reported species. In addition, the translocation of *trnY* in *Parapolybiacrocea* occurs, *trnQ*, *trnM* and *trnY* genes are lost in *Polisteshumilis* mitogenome, and in *Polistesjokahamae* mitogenome, not only *trnD* is in the upstream of *trnK* but also *trnI*, *trnQ* and *trnY* are missing (Figure 2) (Cameron et al. 2008; Song et al. 2016; Peng et al. 2017). In the subfamily Vespinae, except for the incomplete mitogenomes of *Vespulagermanica* and *Vespabicolor*, there is the same rearrangements in other four reported species, such as the translocation of *trnY*, the translocation between *trnQ* and *trnM* genes, between *trnS1* and *trnE* genes, and between *nad1* and *trnL2^(CUN)^* genes, respectively and *Dolichovespulapanda* is different from other four species: the translocation between *trnS1* and *trnE* genes in exchange for shuffling of *trnN* and *trnE* (Figure 2) (Chen et al. 2016; Fan et al. 2017; Kim et al. 2017a; Kim et al. 2017b; Nizar et al. 2017). In general, the rearrangement frequency in Eumeninae is lower than those of both Vespinae and Polistinae. The rearrangement of *tRNAs* is a typical event in the mitogenomes of Hymenoptera (Dowton and Austin 1999; Dowton et al. 2009; Chen et al. 2016).

### Nucleotide composition

To date, the nucleotide compositions of ten complete mitogenomes have been reported in the family Vespidae. In the subfamily Eumeninae, the overall A + T content of *O.a.aterrimus* and *Abispaephippium* mitogenomes is 79.43% and 80.61%, respectively (Table 3). Among all Vespidae mitogenomes, there are no significant differences of the A + T content of Polistinae, i.e., *P.humilis* being 84.73%, *P.jokahamae* 83.41%, and *Parapolybiacrocea* 82.94%, respectively. In the subfamily Vespinae, there are a little differences of the A + T content from *Vespamandarinia* 79.39% to *Dolichovespulapanda* being 84.61%. Generally speaking, the A + T content of Eumeninae is lower than those of both Vespinae and Polistinae. According to these different regions of *O.a.aterrimus* mitogenome, the A + T content of 13 PCGs is 78.27% near to *A.ephippium* (78.67%). In *tRNAs*, *rRNAs*, and CR, the A + T content is 83.41%, 84.29% and 84.69%, respectively. From the A + T content of all known Vespidae complete mitogenomes (Table 3), a universal feature is presumed that A + T content of *tRNAs* and *rRNAs* higher than that of PCGs.

**Table 3.** Nucleotide composition of different regions in all complete Vespidae mitogenomes.

Species	Regions	Size(bp)	A%	T%	G%	C%	(A+T)%	AT-skew	GC-skew
* Orancistrocerus aterrimus aterrimus *	Whole genome	17972	39.53	39.9	8.06	12.51	79.43	-0.005	-0.216
	Protein coding genes	11122	33.15	45.12	10.02	11.72	78.27	-0.153	-0.078
	tRNA genes	1525	42.69	40.72	9.25	7.34	83.41	0.024	0.115
	rRNA genes	2151	41.89	42.4	10.79	4.93	84.29	-0.006	0.373
	Control region	1078	39.8	44.9	6.49	8.81	84.69	-0.06	-0.152
* Abispa ephippium *	Whole genome	16953	39.55	41.05	6.02	13.38	80.61	-0.019	-0.38
	Protein coding genes	11305	35.2	43.48	10.12	11.21	78.67	-0.105	-0.051
	tRNA genes	1787	44.66	38.84	8.95	7.55	83.49	0.07	0.085
	rRNA genes	2180	43.62	38.35	5.14	12.89	81.97	0.064	0.43
	Control region	308	43.83	46.1	1.3	8.77	89.94	-0.025	-0.742
* Polistes jokahamae *	Whole genome	16616	41.97	41.45	5.8	10.79	83.41	0.006	-0.301
	Protein coding genes	10852	36.77	46.61	8.11	8.51	83.38	-0.118	-0.024
	tRNA genes	1318	44.76	42.64	6.98	5.61	87.4	0.024	0.108
	rRNA genes	2257	43.95	41.25	4.3	10.5	85.2	0.032	0.419
	Control region	1096	39.05	46.53	6.84	7.57	85.58	-0.087	-0.051
* Polistes humilis *	Whole genome	14741	43.09	41.65	5.32	9.95	84.73	0.017	-0.303
	Protein coding genes	10852	36.77	46.61	8.11	8.51	83.38	-0.118	-0.024
	tRNA genes	1258	47.22	41.02	6.52	5.25	88.24	0.07	0.108
	rRNA genes	1932	43.27	43.22	9.16	4.35	86.49	0.001	0.356
	Control region	*	*	*	*	*	*	*	*
* Parapolybia crocea *	Whole genome	16619	43.39	39.55	5.91	11.15	82.94	0.046	-0.307
	Protein coding genes	11022	35.48	45.16	9.54	9.82	80.65	-0.12	-0.015
	tRNA genes	1486	44.01	42.13	7.67	6.19	86.14	0.022	0.107
	rRNA genes	2176	40.3	45.96	9.38	4.37	86.26	-0.066	0.365
	Control region	1316	42.25	46.05	5.17	6.53	88.3	-0.043	-0.117
* Vespa ducalis *	Whole genome	15779	40.32	39.8	5.8	14.08	80.12	0.006	-0.417
	Protein coding genes	11159	34.32	43.46	10.36	11.86	77.78	-0.118	-0.067
	tRNA genes	1487	45.46	40.15	8.14	6.25	85.61	0.062	0.131
	rRNA genes	2299	44.58	39.58	11.44	4.39	84.17	0.059	0.445
	Control region	166	46.99	45.78	0	7.23	92.77	0.013	-1
* Vespa mandarinia *	Whole genome	15902	38.88	40.51	6.07	14.53	79.39	-0.021	-0.41
	Protein coding genes	11119	33.73	43.37	10.56	12.35	77.09	-0.125	-0.078
	tRNA genes	1505	45.12	40.47	8.37	6.05	85.58	0.054	0.161
	rRNA genes	1569	43.91	39.64	12.11	4.33	83.56	0.051	0.473
	Control region	200	49	39.5	0.5	11	88.5	0.107	-0.913
* Vespa velutina nigrithorax *	Whole genome	16475	40.3	41.44	5.43	12.83	81.74	-0.014	-0.406
	Protein coding genes	11197	34.99	44.75	9.42	10.83	79.74	-0.122	-0.07
	tRNA genes	1514	44.58	41.35	8.12	5.94	85.93	0.038	0.155
	rRNA genes	2319	45.11	40.06	10.52	4.31	85.17	0.059	0.419
	Control region	132	50.76	41.67	0	7.58	92.42	0.098	-1
* Vespa orientalis *	Whole genome	16101	40.65	40.3	5.86	13.19	80.95	0.004	-0.384
	Protein coding genes	10653	34.5	44.08	9.74	11.68	78.58	-0.122	-0.09
	tRNA genes	1481	45.51	40.51	7.97	6.01	86.02	0.058	0.14
	rRNA genes	2079	43.67	39.15	11.5	5.68	82.83	0.055	0.339
	Control region	60	48.33	41.67	8.33	1.67	90	0.074	0.667
* Dolichovespula panda *	Whole genome	17136	42.8	41.81	5.39	10	84.61	0.012	-0.3
	Protein coding genes	11276	35.82	46.78	8.78	8.62	82.6	-0.133	0.009
	tRNA genes	1506	45.88	40.44	7.9	5.78	86.32	0.063	0.155
	rRNA genes	2126	43.7	40.87	10.68	4.75	84.57	0.033	0.384
	Control region	586	67.24	32.42	0	0.34	99.66	0.349	-1

* *P.humilis* (EU024653), not sequenced for the control region

Two other parameters, AT-skew and GC-skew, have been widely used to measure the nucleotide compositional behaviors of mitogenome in addition to the A + T content (Enrico et al. 2011). The AT skew of *O.a.aterrimus* mitogenome is -0.005 near to 0, and the GC skew (-0.216) is negative. The base composition bias plays an important role in researching the mechanism of replication and transcription of mitogenomes (Wei et al. 2010).

**Figure 3.**
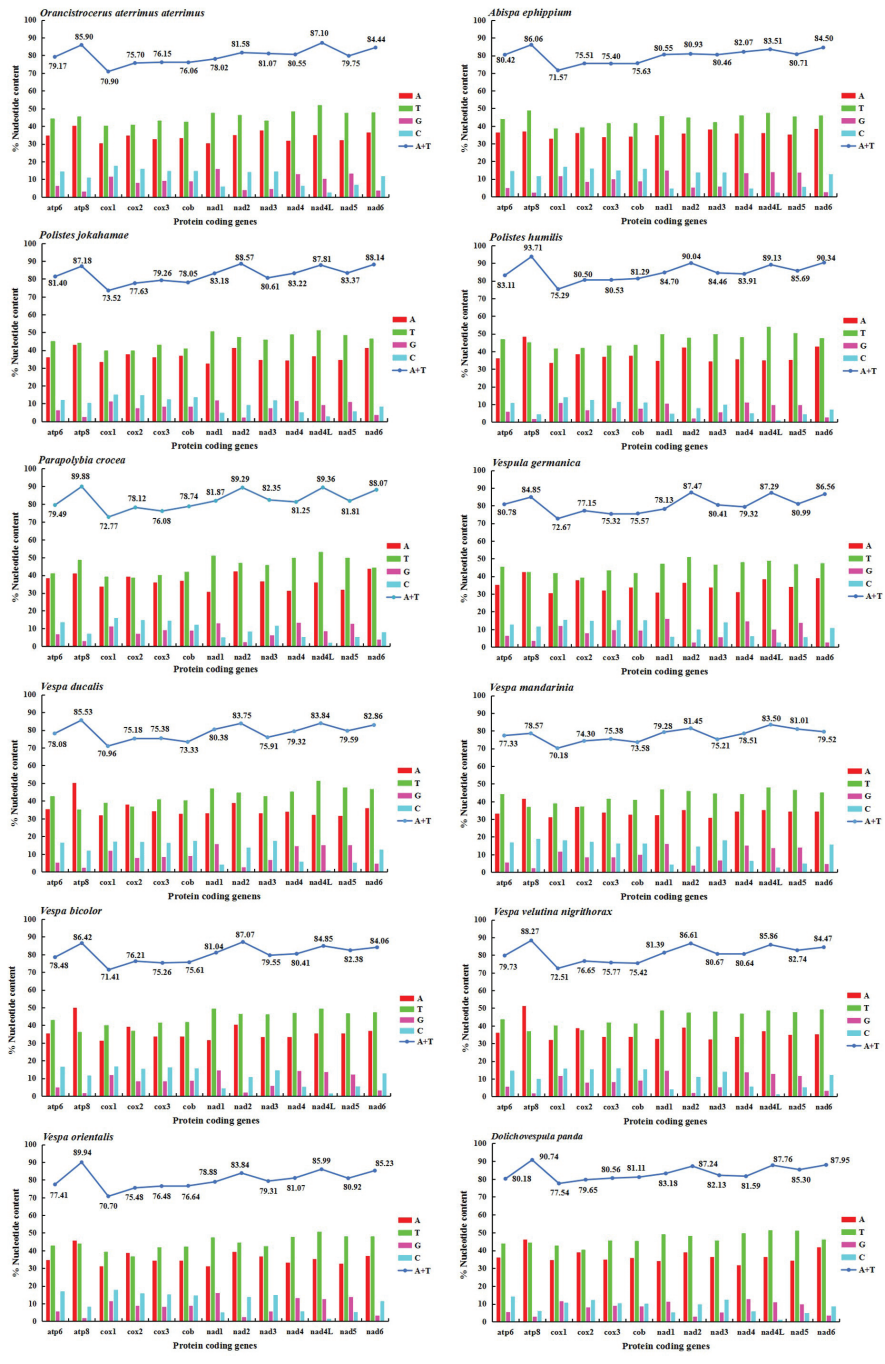
Nucleotide composition of all 13 PCGs of eleven species of Vespidae.

Among the PCGs of 12 Vespidae species (containing two incomplete mitogenomes), the A + T content of *cox1* is the lowest in 13 PCGs, ranging from 70.18% (*Vespamandarinia*) to 75.29% (*P.humilis*) (Figure 3). The A + T content of *atp8*, *nad2*, and *nad4L* is highest (Figure 3). This result ascertains *cox1* is conserved relatively again, which is the reason for former abundant phylogenetic analysis in other insects (Rivera and Currie 2009; Santos et al. 2015). In addition, it is a common phenomenon that T content is more than A, and C content is slightly more than G (Figure 3).

### Protein coding genes

In the 13 PCGs of the *O.a.aterrimus* mitogenome, nine PCGs are encoded in the J-strand, and the other four PCGs are located in the N-strand. The total length of PCGs is 11 122 bp. All PCGs use the conventional start codons ATN except for *cox1* using TTG which was also employed as the initiation codon in other insects (Sheffield et al. 2008; Li et al. 2012a). The termination codons of nine PCGs in *O.a.aterrimus* mitogenome use complete TAA (*nad2, cox1, atp8, atp6, nad3, nad5, nad4l, nad6* and *nad1*), and other four genes have incomplete stop codons T (*cox2, cox3, nad4* and *cytb*). In general, the termination codons of insect mitogenomes PCGs were the TAA or incomplete T (Ojala et al. 1981; Li et al. 2012a).

**Figure 4.**
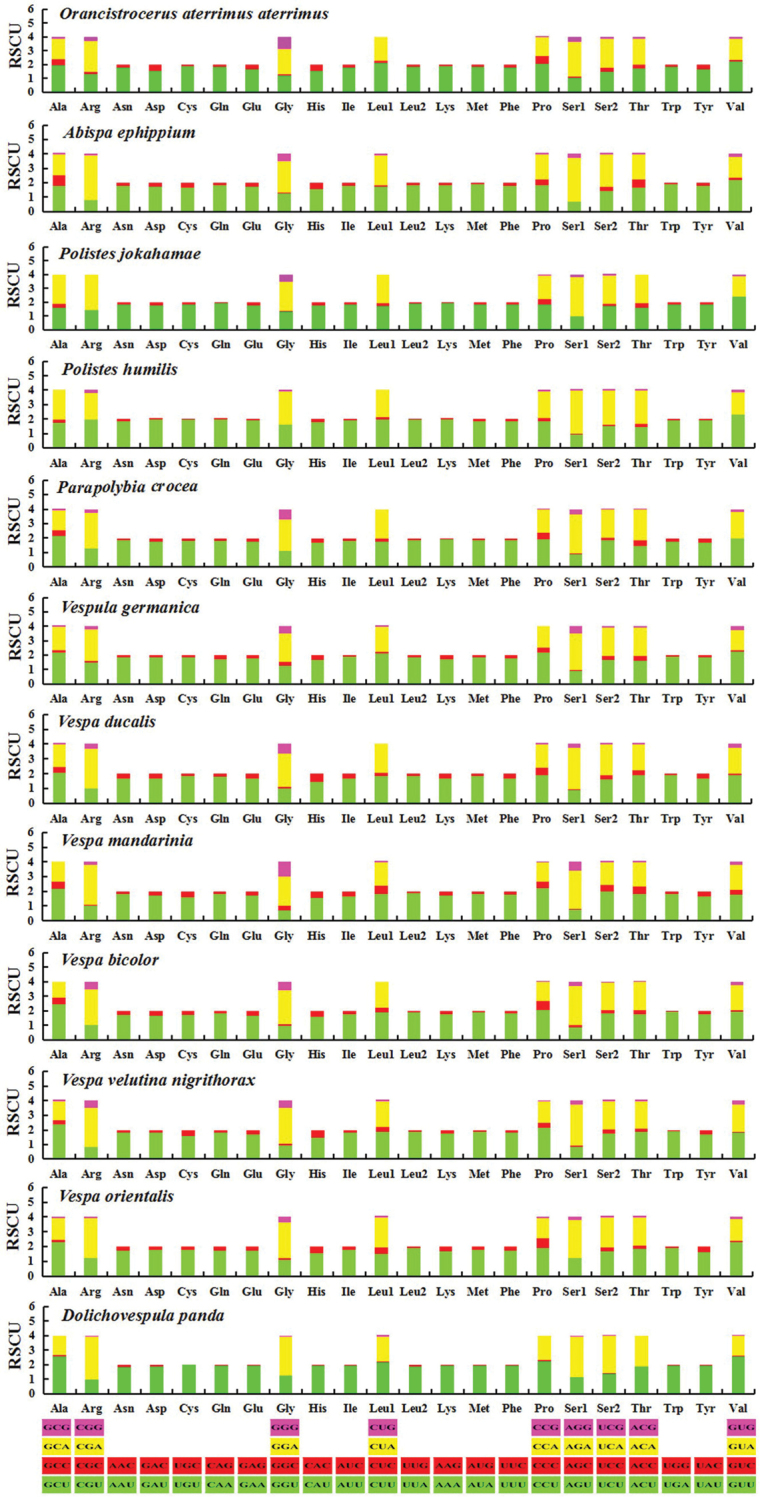
Relative synonymous codon usage (RSCU) in Vespidae. Codon families are displayed along the x-axis.

There is a total of 3697 codons in *O.a.aterrimus* mitogenome, excluding termination codons, which is within the range of the common insect mitogenomes codon number (3585-3746) (Cha et al. 2007). According to the relative synonymous codon usage (RSCU), all of these 12 Vespidae species frequently used UUU, UUA, AUU and AUA (Figure 4), leading to the high A + T content in the PCGs of the family Vespidae mitogenomes. CUG is absent in *O.a.aterrimus* mitogenome and CGC and AGC are absent in *A.ephippium*. Some codons are also lacking in other species of Vespidae. For example, CGC and AGC in *Vespaorientalis*, CUG, GCG, CGC in *V.bicolor* and CCG, ACC, ACG, GCG, UGC, and CGC in *Dolichovespulapanda* are absent, respectively. There are several codons missing in *Polistesjokahamae*, namely, CUG, GUC, ACG, GCG, CGC, CGG, AGC; and CUG, GUC, GCG, CGC, and GGC are also lacked in *P.humilis* (Figure 4). Thus, the amount of absent codons in Vespinae and Polistinae is more than in Eumeninae.

### Transfer RNA and ribosomal RNA genes

**Figure 5.**
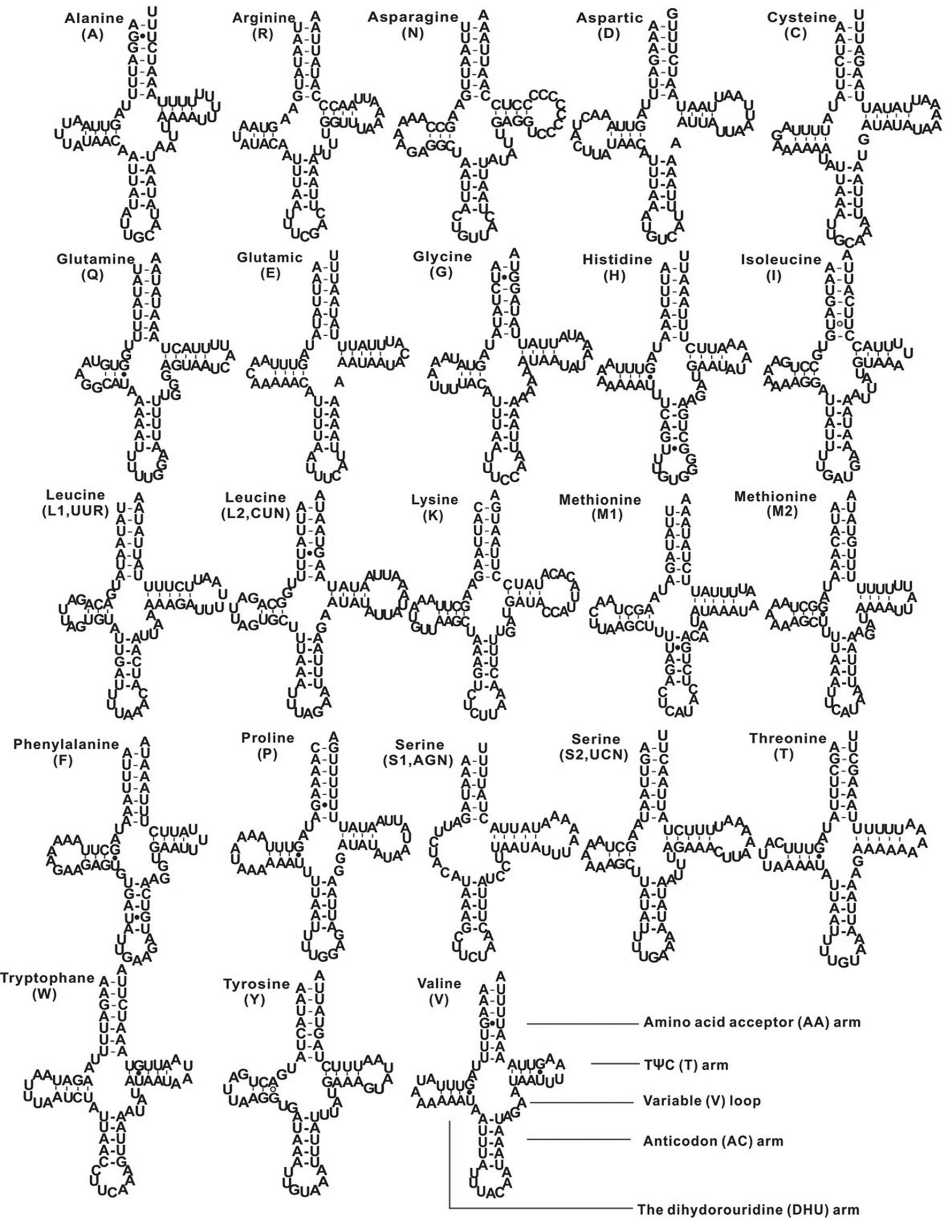
Secondary structures of 23 tRNAs of *O.a.aterrimus* mitochondrial genome. Watson-Crick bonds are showed by dashes, GU pairs by filled dots, and AG and UU by open dots.

There are 23 tRNAs found in *O.a.aterrimus* mitogenome and their lengths range from 60 bp (*trnS1*) to 72 bp (*trnK*) including an extra *trnM2*, whereas usually there are 22 tRNAs in other insects (Boore 1999; Chen et al. 2015). Among 23 anticodons of these tRNAs, 21 are coincident with the majority of insects mitogenomes (Lee et al. 2008; Hua et al. 2016), but *trnI* and *trnS1* change from CCT to GAT, and GCT to TCT, respectively. Except for *trnS1*, the other 22 tRNAs have the capability of folding into typical clover-leaf secondary structures. The secondary structure of *trnS1* lacks the dihydrouridine DHU arm and reduces its shape to a simple loop (Figure 5), which is a common phenomenon in metazoan mitogenomes (Wolstenholme 1992; Li et al. 2012b). There are 20 mismatches in 13 tRNAs, including 18 unmatched GU base pairs, an unmatched AG, and an unmatched UU (Figure 5).

The length of *rrnL* is 1 363 bp long, located between *nad1* and *trnV*, and *rrnS* 788 bp long in minority strand between *trnV* and CR. The A + T content of two genes is 84.29% (*rrnL* and *rrnS*) (Table 3).

### A control region and a non-coding region

The CR plays an important role in regulating of replication and transcription of mitogenomes (Taanman 1999; Saito et al. 2005). The CR of *O.a.aterrimus* mitogenome is 1078 bp long, located between *rrnS* and *trnI*. The A + T content of this region (84.69%) is higher than other region of the *O.a.aterrimus* mitogenome. There is a tandem repeat model of 28 bp (TATTCCATTTAAGTTCGTAAAAACTAAT) which occurs more than eight times in the *O.a.aterrimus* mitogenome. Tandem repeat structures in the CR are different in different species (Peng et al. 2017). There is also a poly-T stretch of 13 bp, which may be as recognition site for the initiation of replication in the mitogenomes (Andrews et al. 1999). In the *O.a.aterrimus* mitogenome, a NCR is situated in position 128 - 2 073 (1 946 bp) between *trnM1* and *trnQ*, which is reported in most insect mitogenomes (Saito et al. 2005; Cameron et al. 2008; Jiang et al. 2016). The A + T content of NCR is 73.69%, among which there is 97 bp (close to *trnQ* gene) with obviously high A + T content 90.72%. In addition, two tandem repetitive sequences are found in the NCR, which repeated 17 and 18 times, respectively.

### Phylogenetic relationships

**Figure 6.**
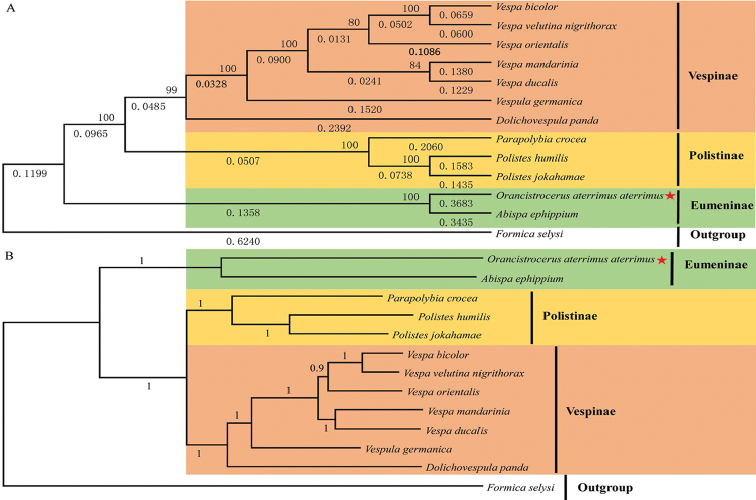
The phylogenetic relationships were established by the 13 PCGs using ML (**A**) and BI (**B**) methods. Numbers abutting branches were bootstrap percentages with 1000 replicates (**A**) and Bayesian posterior probabilities (**B**). Red pentagram refers to the mitogenome sequences of *O.a.aterrimus*.

The best fitting model GTR + G + I was selected for ML analysis. The phylogeny of mitogenomes in Vespidae was constructed based on the nucleotide sequences of 13 PCGs of 13 species using ML and BI methods (Figure 6). The phylogenetic relationships between 12 species in the family Vespidae are (((((*Vespabicolor* + *Vespavelutinanigrithorax*) + *Vespaorientalis*) + (*Vespamandarinia* + *Vespaducalis*)) + *Vespulagermanica*+ *Dolichovespulapanda*) + (*Parapolybiacrocea*+ (*Polisteshumilissynoecus* + *Polistesjokahamae*))) + (*Orancistrocerusaterrimusaterrimus* + *Abispaephippium*) (Figure 6). *O.a.aterrimus* and *A.ephippium* belong to the subfamily Eumeninae, which is concordant with morphological classification. In the present study, Eumeninae is the sister group with (Polistinae + Vespinae), which is different from morphological classification “(Eumeninae + (Stenogastriinae + (Vespinae + Polistinae))) ” (Carpenter 1982, 2003). So far, there is no reported mitogenome in the subfamily Stenogastriinae, so the relationships among the four subfamilies Eumeninae, Stenogastriinae, Vespinae and Polistinae based on mitogenomes need to be further explored in our follow-up studies.

## Conclusions

According to nine complete mitogenomes reported in the family Vespidae, gene numbers of two species (38 and 41 genes) of the subfamily Eumeninae are more than those of the other seven species (34 - 37 genes) of both Polistinae and Vespinae. The rearrangements of tRNAs are common in Vespidae, but rearrangement rules are different in different subfamilies. The translocation between trnS1 and trnE only happens in the subfamily Vespinae, and there are the same rearrangements in these four complete mitogenomes of *Vespamandarinia*, *V.ducalis*, *V.orientalis*, and *V.velutinanigrithorax*. The translocation of *trnY* occurs in both Vespinae and Polistinae, whereas *trnY* location in Eumeninae is consistent with that of the sawfly *Pergacondei*. The number of absent codons in Eumeninae is less than Vespinae and Polistinae. The phylogenic results of mitogenomes show that *O.a.aterrimus* and *Abispaephippium* belong to Eumeninae and (Polistinae + Vespinae) and Eumeninae constitute a sister group. Lastly, these results of this study might suggest that Eumeninae derived earlier than both Polistinae and Vespinae, which is consistent with reported research based on morphology.
